# Index and targeted community‐based testing to optimize HIV case finding and ART linkage among men in Zambia

**DOI:** 10.1002/jia2.25520

**Published:** 2020-06-26

**Authors:** Linah K Mwango, Kristen A Stafford, Natalia C Blanco, Marie‐Claude Lavoie, Morley Mujansi, Nasho Nyirongo, Kalima Tembo, Henry Sakala, Julian Chipukuma, Beauty Phiri, Carol Nzangwa, Susan Mwandila, Kennedy C Nkwemu, Ahmed Saadani, Annie Mwila, Michael E Herce, Cassidy W Claassen

**Affiliations:** ^1^ Maryland Global Initiatives Corporation Zambia Lusaka Zambia; ^2^ Center for International Health, Education, and Biosecurity University of Maryland School of Medicine Baltimore MD USA; ^3^ Institute of Human Virology University of Maryland School of Medicine Baltimore MD USA; ^4^ Department of Epidemiology and Public Health University of Maryland School of Medicine Baltimore MD USA; ^5^ U.S. Center for Disease Control and Prevention Lusaka Zambia; ^6^ Institute for Global Health and Infectious Diseases University of North Carolina School of Medicine Chapel Hill NC USA; ^7^ Centre for Infectious Disease Research in Zambia (CIDRZ) Lusaka Zambia

**Keywords:** index testing services, finding men, HIV care continuum, linkage to care, HIV testing, sub‐Saharan Africa, differentiated care

## Abstract

**Introduction:**

Current healthcare systems fail to provide adequate HIV services to men. In Zambia, 25% of adult men living with HIV were unaware of their HIV status in 2018, and 12% of those who were unaware of their HIV statu were not receiving antiretroviral therapy (ART) due to pervasive barriers to HIV testing services (HTS) and linkage to ART. To identify men and key and priority populations living with HIV in Zambia, and link them to care and treatment, we implemented the Community Impact to Reach Key and Underserved Individuals for Treatment and Support (CIRKUITS) project. We present HTS and ART linkage results from the first year of CIRKUITS.

**Methods:**

CIRKUITS aimed to reach beneficiaries by training, mentoring, and deploying community health workers to provide index testing services and targeted community HTS. Community leaders and workplace supervisors were engaged to enable workplace HTS for men. To evaluate the effects of these interventions, we collected age‐ and sex‐disaggregated routinely collected programme data for the first 12 months of the project (October 2018 to September 2019) across 37 CIRKUITS‐supported facilities in three provinces. We performed descriptive statistics and estimated index cascades for indicators of interest, and used Chi square tests to compare indicators by age, sex, and district strata.

**Results:**

Over 12 months, CIRKUITS tested 38,255 persons for HIV, identifying 10,974 (29%) new people living with HIV, of whom 10,239 (93%) were linked to ART. Among men, CIRKUITS tested 18,336 clients and identified 4458 (24%) as HIV positive, linked 4132 (93%) to ART. Men who tested HIV negative were referred to preventative services. Of the men found HIV positive, and 13.0% were aged 15 to 24 years, 60.3% were aged 25 to 39, 20.9% were aged 40 to 49 and 5.8% were ≥50 years old. Index testing services identified 2186 (49%) of HIV‐positive men, with a positivity yield of 40% and linkage of 88%. Targeted community testing modalities accounted for 2272 (51%) of HIV‐positive men identified, with positivity yield of 17% and linkage of 97%.

**Conclusions:**

Index testing and targeted community‐based HTS are effective strategies to identify men living with HIV in Zambia. Index testing results in higher yield, but lower linkage and fewer absolute men identified compared to targeted community‐based HTS.

## 1 INTRODUCTION

Of the 37.9 million people living with HIV (PLHIV) globally, including the 25.6 million residing in sub‐Saharan Africa (SSA), 21% remain unaware of their HIV status [1]. To address this gap in SSA, efficient, targeted and evidence‐based approaches are urgently needed for HIV case finding programmes, particularly for populations less likely to know their status, such as men [1]. Traditional approaches to HIV testing, such as facility‐based testing, while effective for identifying HIV‐positive adult women in SSA, have yielded sub‐optimal results for reaching men [2].

The HIV epidemic in Zambia is generalized, with HIV prevalence standing at 11.1% among adults aged 15 to 49 [3]. Among men, awareness of HIV status is substantially lower than among women, with 25% of Zambian men having not been tested for HIV and received their results, compared to only 15% of adult women [3]. The problem is most acute among younger men, with 67.1% of HIV‐positive males 25 to 29 years old reporting being unaware of their HIV status [4]. Compared to women, men in Zambia test and obtain their results less frequently and are less likely to link to care [3, 4]. Indeed, in the HPTN 071 PopART trial in Zambia, the groups most likely to be missing from the testing and linkage cascade were men and youth [5].

Multiple factors contribute to low testing and linkage rates among men in the HIV care cascade, including cultural notions of masculinity, but also structural factors such as clinic hours and labour requirements [6, 7]. Men also cite fears of discovering their HIV status [8] and also often test by proxy and rely on their wives’ HIV results [6].

Available facility‐based HIV testing modalities in Zambia include voluntary counselling and testing (VCT) and provider‐initiated testing and counselling (PITC), which have remained relatively low‐yield in SSA [9]. Most HIV programmes in Zambia reported positivity yields in VCT and PITC departments of approximately 4% to 8% [10], with the highest yield reported from facilities in Zambia at 9% [11]. Community‐based HIV testing modalities include VCT and mobile/outreach, as well as index testing services (ITS) and partner notification services (PNS) for index clients, which was introduced in Zambia in 2017 to improve HIV case identification.

Community‐based HIV testing in SSA has been associated with HIV positivity yields of 6% to 11%, according to a meta‐analysis of 126 HIV testing studies in SSA [12]. Targeting community‐based testing in venues that feature characteristics associated with a low “number needed to test” to identify a new HIV case, such as where people meet new sex partners, is a promising strategy to improve testing yield [13]. In Zambia, community‐based HIV positivity yields in men ranges from 5.2 to 5.7% [14, 15] compared to 9.3% among women [14].

By comparison, index testing has consistently been demonstrated to be an effective model for HIV case identification in SSA. Across PEPFAR‐supported countries during early ITS rollout in 2016 to 2018, index testing positivity was 9.8% [9]. Recent literature points to higher yields from index testing in the general population: 10.3% in South Africa [16], 21‐29% in Kenya [17], and 32.6% and 38% positivity in similar programs in Zimbabwe [18] and South Africa [19], respectively. At the highest range of programmatic reports, yield in Nigeria [20] and Cameroon [21] reached 51% and 51.8% respectively. ITS has also been shown to increase HIV positivity yield among men in the region [18], as have targeted community HIV testing modalities such as mobile and workplace testing [22].

The Community Impact to Reach Key and Underserved Individuals for Treatment and Support (CIRKUITS) project, implemented by the Center for International Health, Education, and Biosecurity (Ciheb) at the University of Maryland Baltimore (UMB) and the Centre for Infectious Disease Research Zambia (CIDRZ) aimed to enhance HIV case finding, linkage to care and treatment and adherence support at the community level in Zambia. The objective of this paper was to present the CIRKUITS approach to case finding and examine HIV positivity yield and antiretroviral therapy (ART) linkage across index testing and targeted community testing modalities, with sub‐analyses by sex, age groups and districts.

## 2 METHODS

### 2.1 Study design and setting

**Figure 1:**
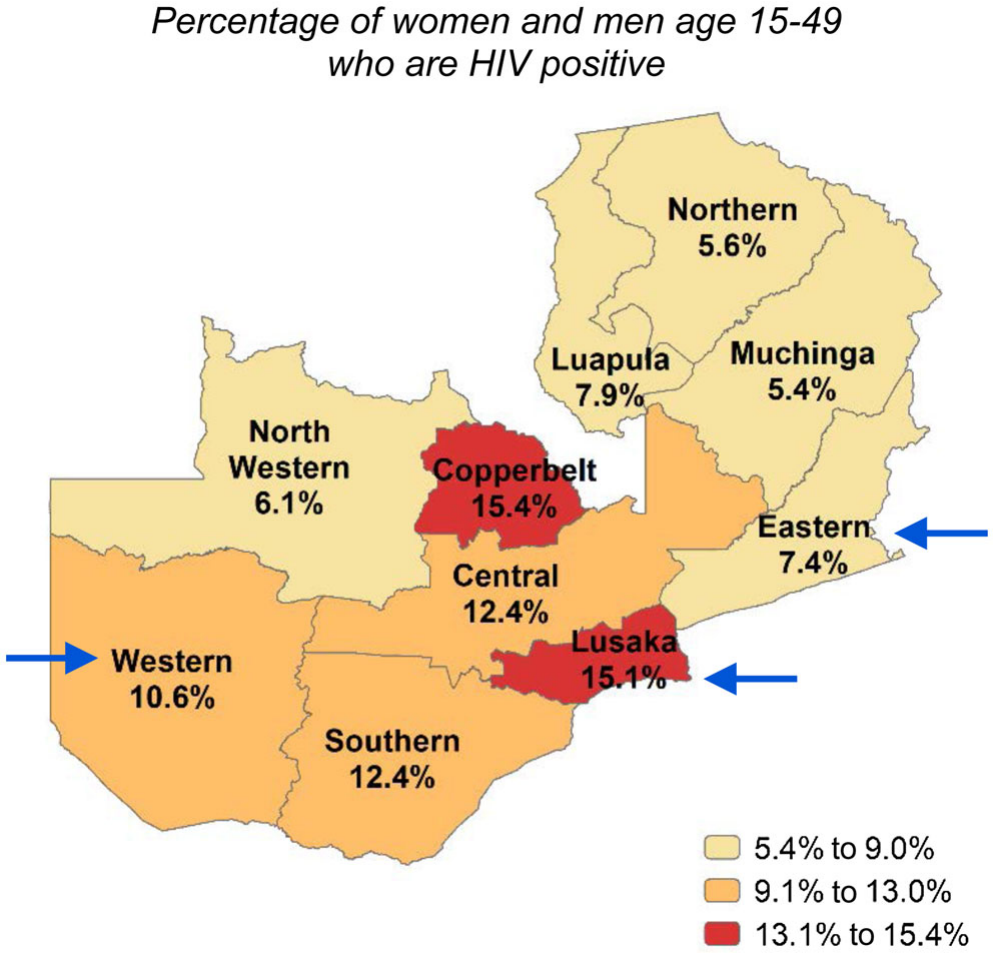
Map of Zambia with HIV prevalence among adults aged 15 to 49 by province. Arrows indicate the provinces in which CIRKUITS was implemented. Data from ZDHS 2018 [3].

We conducted a retrospective analysis of programmatic aggregate data collected from 1 October 2018 to 30 September 2019 as part of routine CIRKUITS service delivery. CIRKUITS was implemented across three of Zambia’s 10 provinces, Eastern, Western and Lusaka, where estimated HIV prevalence was 7.4%, 10.6%, and 15.1%, respectively (Figure 1) [4]. CIRKUITS was implemented in communities within the catchment areas of 37 participating facilities in these provinces: four urban and five rural facilities in Eastern; three urban and 11 rural facilities in Western and 14 urban facilities in Lusaka. HIV services were delivered to residents in the community by community health workers (CHWs) supervised by community liaison officers (CLOs) working with Ministry of Health (MOH) staff in the corresponding health facilities.

### 2.2 Study population

CIRKUITS provided HIV prevention education, testing services and facilitated ART linkage services for all individuals in the community who may be at risk for HIV. In this analysis, we included all individuals 15 years or older that received any community‐based HIV testing service as part of the CIRKUITS programme.

### 2.3 CIRKUITS intervention

The CIRKUITS approach centred around the work of trained CHWs who were supervised and mentored by CLOs to offer HIV prevention and testing services in the community. CHWs offered different HIV testing services (HTS) modalities to individuals in the community, including voluntary ITS and targeted community testing services. They also provided a combination of HIV preventive services, including health education and referral for voluntary medical male circumcision (VMMC), HIV pre‐exposure prophylaxis (PrEP), family planning (FP) and condom distribution. The CLOs and CHWs were supervised by the public health facility staff in their catchment area, were assessed and certified as competent in ITS and PNS by MOH and CIRKUITS technical staff, and reported all community activities to the facility. CIRKUITS had a bidirectional referral system in which either facility staff, or CHWs and CLOs, could direct clients to health services based on their medical conditions and preferences. During the study period, CIRKUITS trained, mentored and deployed 157 CHWs and 22 CLOs to conduct different community‐based testing modalities, including ITS (Table 1).

**Table 1:** CIRKUITS staffing across supported provinces and districts

Province	Estimated provincial HIV prevalence^a^	Provincial nurses	District	District CLOs	CIRKUITS‐supported facilities	CHWs in district
Eastern	7.4%	1	Chipata	2	6	20
			Petauke	2	3	39
Western	10.6%	1	Mongu	3	7	20
			Limulunga	3	7	15
Lusaka	15.1%	2	Lusaka Urban	12	14	63
Total	Country: 11.1%	4	5	22	37	157

CIRKUITS, Community Impact to Reach Key and Underserved Individuals for Treatment and Support; CLO, community liaison officer; CHW, community health worker.

^a^ Prevalence data from ZDHS 2018 [3].

### 2.4 CIRKUITS approach to index testing and partner notification services

**Figure 2:**
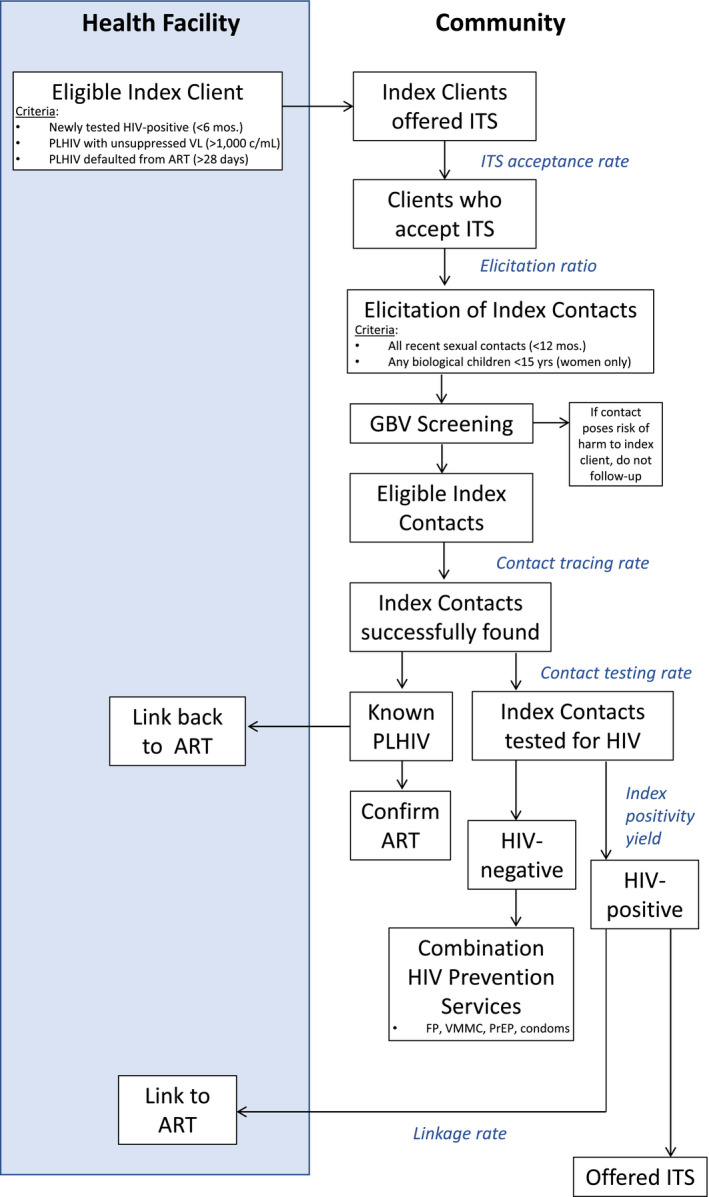
CIRKUITS (Community Impact to Reach Key and Underserved Individuals for Treatment and Support) steps for index testing. Boxes in shaded area represent facility‐based activities; other boxes represent activities taking place in the community. Text in blue italics reflects relevant programme metrics. These steps were initially developed by UMB, and then adopted in the National Zambia Consolidated Guidelines for Prevention and Treatment of HIV Infection 2020 [25]. FP, family planning; GBV, gender‐based violence; ITS, index testing services; PLHIV, people living with HIV; PrEP, pre‐exposure prophylaxis; VMMC, voluntary medical male circumcision.

CIRKUITS employed voluntary and confidential ITS with PNS to increase HIV case identification in the community (see Figure 2). The facility staff, together with CLOs and CHWs, identified individuals as potential index clients eligible for ITS. These included individuals who were diagnosed with HIV in the past six months, had an unsuppressed viral load (>1000 copies/mL), and/or were lost to follow up from ART (>28 days elapsed since last pharmacy pickup). CLOs and CHWs contacted eligible index clients and offered ITS. If the client consented to these services, the CHW then counselled the client on the benefits and importance of their contacts knowing their HIV status, and then proceeded to elicit all sexual contacts in the past year; if the index client was female, they also elicited biological children <15 years. For clients who consented to PNS, the health provider and the client agreed on a referral process for the index contacts following the WHO‐recommended approach [23]. CIRKUITS offered three approaches for index client partner notification: (i) for client referral, the index client chose to disclose their HIV status to sexual partners and suggest HIV testing; (ii) for provider referral, CLOs or CHWs obtained consent from the index client to contact sexual partners or biological children to offer HIV testing; (iii) for dual referral, the CHWs or CLOs accompanied the index client to assist with the disclosure of HIV status and offer HTS to their partner(s) or biological children. To minimize gender‐based violence (GBV) stemming from partner notification services during index testing, all index cases were screened for risk of GBV via a MOH‐approved GBV screening tool. CHWs did not trace any index contact posing a risk of GBV to the index client. Individuals experiencing GBV or at risk for GBV were accompanied to “GBV One Stop Centres,” a structured system in Zambia, where clinical, legal and psychosocial counsellors provide comprehensive care to GBV clients [24].

The CHWs then traced all eligible index contacts in the community and offered voluntary and confidential HTS. Sexual partners that tested HIV negative were offered combination HIV prevention services, including FP, VMMC and condoms. High‐risk adolescents were linked to youth‐friendly spaces established at the health facilities, as well as DREAMS safe spaces in the two DREAMS‐supported districts (Lusaka and Chipata). People identified at risk of HIV according to the MOH screening tool were also referred for PrEP. Index contacts identified with HIV were actively accompanied to the facility by the CHW to initiate ART. Any client that deferred same‐day ART was followed up in the community for later ART initiation.

### 2.5 CIRKUITS targeted community‐based testing modalities

In addition to ITS, CIRKUITS provided other community‐based testing modalities, including testing in mobile, temporary or other outreach locations (community centres, schools, workplaces, tents) and standalone VCT centres situated outside of affiliated health facilities. The locale of these testing sites was chosen to target priority populations, and often included health education via drama and music. Clients were educated about HIV prevention and the benefits of knowing their HIV status and then offered HTS. HIV‐negative persons were offered combination HIV prevention services, including condoms, FP, VMMC and PrEP (if appropriate). HIV‐positive persons were offered same‐day ART in the community. Alongside testing services, a clinical team was present to initiate ART immediately, including HIV nurse prescribers, pharmacists and psychosocial counsellors. In cases where no facility healthcare workers were available to join the clinical team due to competing facility demands, clients were actively escorted to the facilities by a CLO or CHW for ART initiation, followed by peer health navigation and adherence support to encourage sustained ART.

### 2.6 Testing and linkage strategies to find and link men

To optimize the uptake and coverage of HIV testing among men, CIRKUITS trained CLOs and CHWs on specific strategies to reach men with HTS and health promotion and education, such as where to find men, how to provide tailored messaging for men on HIV/AIDS, and how to conduct pre‐ and post‐counselling for men. CIRKUITS conducted HIV prevention and HTS sessions in workplaces that predominantly employ men, such as construction sites, breweries and bottling companies, police departments, and security, electricity and utility companies. This was followed by orientation of human resource managers in the companies on the benefits of HIV prevention and testing services. This initiative resulted in development of workplace policies promoting routine HIV testing that resulted in the CIRKUITS team being invited once a month to provide HIV prevention and testing services. All HTS services were conducted in a confidential manner according to national guidelines [25]. CIRKUITS also worked with local municipal councils and business leaders, such as marketplace chairmen and bus station managers, to deliver HTS services in male‐dense areas such as markets, taxi ranks, car washes and bus stations. As a cross‐cutting strategy, CIRKUITS partnered with traditional and religious leaders such as chiefs, village headmen and pastors to deliver orientation trainings on HIV/AIDS and encourage community‐based HTS. These leaders became instrumental in mobilizing and organizing community HTS activities at sites where men congregate, including *insakas* (meetings of village elders), churches and community sporting events. In addition, CIRKUITS worked with facilities to expand male‐focused services, including provision of male‐oriented sexual reproductive health services such as prostate cancer screening, sexually transmitted infection screening and treatment and condom pick‐up points, as well as extension of clinic hours during weekdays and weekends to offer more flexibility and convenience for male clients.

### 2.7 Outcomes and variables

Outcomes of interest included: (1) positivity yield, defined as the number of individuals identified HIV positive divided by the total number of individuals who received HTS and received their test results; and (2) linkage rate, defined as the total number of individuals newly enrolled on ART divided by the number of individuals identified as HIV positive. We also developed an index testing cascade comprised of the following variables: (1) number of index cases offered ITS; (2) number of index cases that accepted ITS; (3) number of contacts elicited; (4) number of contacts tested; (5) number of contacts identified HIV positive and (6) number of HIV‐positive clients linked to ART, defined as number of individuals newly enrolled on ART during the evaluation period. We also calculated the elicitation ratio, defined as the number of contacts elicited per index patient.

For testing modalities, we compared community index testing to other targeted community testing modalities, which included mobile and community‐based testing locations (community centres, schools, workplaces, tents) and standalone VCT. For outcomes, testing modalities and age groups disaggregation, we used definitions and guidance from the PEPFAR Monitoring, Evaluation and Reporting Indicator Reference Sheet (MER) version 2.3 [26].

### 2.8 Data sources and collections

Aggregated data were abstracted from routine Zambian MOH HTS, index testing and linkage registers. At community level, each CHW collected patient‐level information onto individual community HTS and index elicitation forms. At facility level, CLOs then merged these data and entered them into facility registers. Community data was also manually entered into the UMB electronic Community Information Management System (eCOMMIS), a customized web‐based District Health Information Software 2 (DHIS2) data platform. DHIS2 is a health management information system (HMIS) platform commonly used in low and middle‐income countries [27]. eCOMMIS ensured data quality relevant to several programmatic elements, included training, supervision, weekly data reviews and validation.

### 2.9 Statistical analysis

Proportions were calculated for outcomes of interest, and the index testing cascade analysed using descriptive statistics. Chi‐square tests were used to compare overall positivity yield and ART linkage by age, sex and district strata, as well as differences between HIV positivity yield by testing modalities across sex and age strata. Chi‐square tests were also used to compare the proportion of index testing acceptance, contact tracing, positivity and ART linkage along the index cascade by sex. All analyses were performed using SAS 9.4 (Cary, NC).

### 2.10 Ethical approval

Ethical approval for this retrospective analysis of aggregate routine programme data was covered by the routine PEPFAR MER data protocol approved by the ERES Converge Zambian Institutional Review Board (IRB), the Zambian National Health Research Authority, the CDC, and the UMB IRB.

## 3 RESULTS

### 3.1 CIRKUITS HIV testing and linkage services (all community modalities)

A total of 38,255 individuals 15 years or older were tested for HIV from 1 October 2018 to 30 September 2019 (Table 2). Among these, 18,336 (47.9%) were male and 19,919 (52.1%) female. Forty‐five percent of HIV testing was performed among individuals aged 20 to 29 years and 51.9% occurred in Lusaka District.

**Table 2:** Overall CIRKUITS HIV testing performance from October 2018 to September 2019

	HIV testing services (n)	Persons tested HIV positive (n)	Positivity yield (%)	*p* value	Persons linked to ART (n)	ART linkage^a^ (%)	*p* value
Sex				<0.001			0.033
Male	18336	4458	24.3		4132	92.7	
Female	19919	6516	32.7		6107	93.7	
Age bands, years				<0.001			<0.001
15 to 19	3968	516	13.0		476	92.2	
20 to 24	8686	2049	23.6		1850	90.3	
25 to 29	8361	2413	28.9		2286	94.7	
30 to 34	6334	2206	34.8		2076	94.1	
35 to 39	4737	1741	36.8		1645	94.5	
40 to 44	3153	1022	32.4		943	92.3	
45 to 49	1628	587	36.1		550	93.7	
50+	1388	440	31.7		413	93.9	
Districts				<0.001			<0.001
Chipata	6037	1354	22.4		1289	95.2	
Limulunga	2882	629	21.8		606	96.3	
Lusaka	19857	6374	32.1		5868	92.1	
Mongu	5311	1586	29.9		1495	94.3	
Petauke	4168	1031	24.7		981	95.2	
Total	38255	10974	28.7		10239	93.3	

ART, antiretroviral therapy; CIRKUITS, Community Impact to Reach Key and Underserved Individuals for Treatment and Support.

^a^ This corresponds to the TX_NEW proxy measure.

Among those tested, 10,974 were identified as HIV positive, corresponding to an overall positivity yield of 28.7% (Table 2). Both the absolute numbers of individuals tested positive (4458 vs. 6516) and the positivity yield (24.3% vs. 32.7%) were higher among women than men (*p* < 0.001). Across age groups, positivity yield ranged between 13.0% and 36.8% (*p* < 0.001), peaking among people aged from 35 to 39. Positivity yield ranged between 21.8% and 32.1% across districts (*p* < 0.001), with the highest yield in Lusaka.

Of the 10,974 identified PLHIV, 10,239 (93.3%) were linked to ART (Table 2). Linkage to ART differed significantly by gender (*p* = 0.033). Linkage to ART ranged between 90.3% and 94.7% across age groups and between 92.1% and 96.3% across districts (*p* < 0.001).

### 3.2 CIRKUITS HIV positivity yields and contributions by testing modality

A total of 11,762 contacts of index clients were tested through index testing, of whom 5260 were HIV positive, for a positivity yield of 44.7% (Table 3). Both the absolute numbers of people tested (6277 for women and 5485 for men) and the positivity yield were higher among women (49.0%) compared to men (39.9%). Across age groups, positivity yields ranged from 29.3% to 50.9%, peaking across people aged 35 to 39 years. Among the 10,974 individuals diagnosed with HIV in the CIRKUITS project, 5260 were identified via index testing, for a contribution to overall HIV positives identified of 47.9%.

**Table 3:** HIV positivity yields by testing modality from October 2018 to September 2019

	Index testing			Other community testing			Difference^a^
	No. tested	No. positives	Yield (%)	No. tested	No. positives	Yield (%)	
Sex							
Male	5485	2186	39.9	12851	2272	17.7	22.2
Female	6277	3074	49.0	13642	3442	25.2	23.7
Age bands, years							
15 to 19	669	196	29.3	3299	320	9.7	19.6
20 to 24	2030	858	42.3	6656	1191	17.9	24.4
25 to 29	2723	1174	43.1	5638	1239	22.0	21.1
30 to 34	2305	1063	46.1	4029	1143	28.4	17.7
35 to 39	1826	929	50.9	2911	812	27.9	23.0
40 to 44	1150	539	46.9	2003	483	24.1	22.8
45 to 49	595	294	49.4	1033	293	28.4	21.0
50+	464	207	44.6	924	233	25.2	19.4
Overall	11762	5260	44.7	26493	5714	21.6	23.2

^a^ All differences were statistically significant at *p* < 0.001.

Through the other targeted community‐based testing modalities combined, a total of 26,493 individuals were tested, of whom 5714 were HIV positive, for a positivity yield of 21.6% (Table 3). Both the absolute numbers tested (12,851 vs. 13,642) and positivity yield (17.7% vs. 25.2%) were higher for females than males. Across age groups, positivity yields ranged between 9.7% and 28.4%, peaking across people aged 30‐34 and 45‐49. Non‐index community testing modalities contributed 52.1% of overall HIV‐positive clients identified by CIRKUITS.

The overall positivity yield between the two categories of testing modalities was significantly different (*p* < 0.001), where index testing had a higher positivity yield than the other community testing modalities. These differences remain when compared by age and sex strata (Table 3).

### 3.3 Index testing cascade

**Figure 3:**
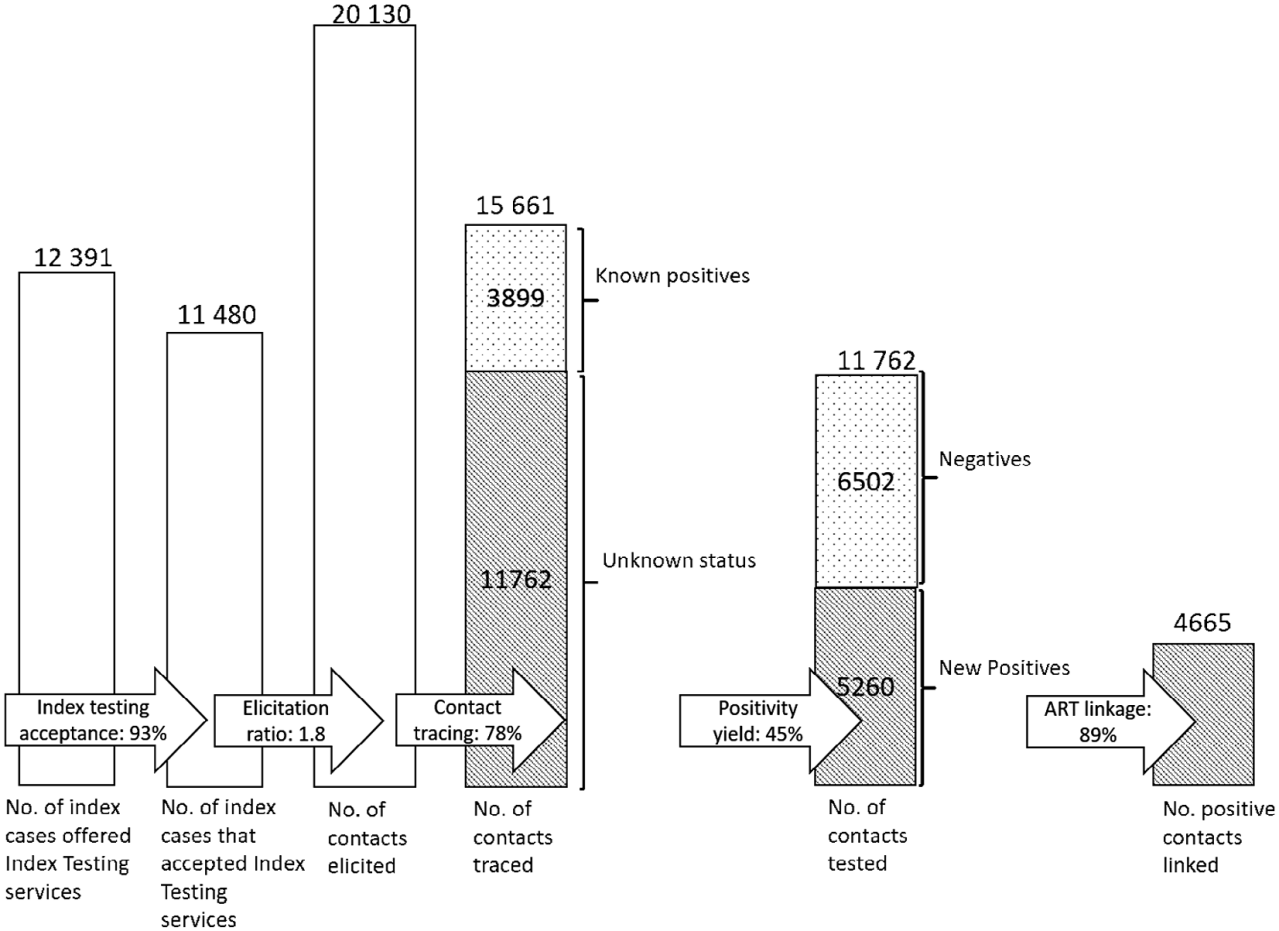
Overall index testing cascade.

CIRKUITS offered ITS to 12,391 HIV‐positive clients, including the 10,974 newly identified HIV‐positive clients from community HTS modalities and 1417 PLHIV found to have an unsuppressed viral load. Among the 12,391 HIV‐positive clients, 11,480 individuals accepted index testing services, for an overall 92.6% index testing acceptance. Acceptance rate did not significantly differ by sex (88.4% for females vs. 89.2% for males, *p* = 0.159). A total of 20,130 contacts were elicited from 11,480 individuals who accepted index testing services, for an elicitation ratio of 1:1.8. A total of 15,661 of the elicited contacts were traced, for a contact tracing rate of 77.8%. Contact tracing rate was higher among females (81.8%) than males (73.7%) (*p* < 0.001). Among the 15,661 traced contacts, 160 (1.0%) were known HIV‐positive clients who were not on ART, 3739 (23.9%) were known HIV‐positive clients who were on ART, and 11,762 (75.1%) were contacts with unknown HIV status. All traced contacts with unknown status were tested for HIV; of these, 5260 were newly diagnosed as HIV positive, representing 44.7% positivity yield (Figure 3). Among the individuals diagnosed with HIV, 2244 (42.7%) were women 25 years or older, 1962 (37.3%) were men 25 years or older, 830 (15.8%) were AGYW ages 15 to 24 and 224 (4.2%) were adolescent males ages 15 to 24. Positivity yield was higher among females (49.0%) than males (39.9%) (*p* < 0.001). Among the 5260 newly diagnosed HIV‐positive clients, 4665 were linked to ART for an overall ART linkage rate of 88.7%. Females had an ART linkage rate of 89.2% and males had an ART linkage of 87.9% (*p* = 0.140).

### 3.4 Male HTS

Over a 12‐month period, a total of 18,336 males were tested and 4458 were identified as HIV positive for an overall positivity yield of 24.3%. Of men identified as HIV positive, 13.0% were aged 15 to 24, 60.3% were aged 25 to 39, 20.9% were aged 40 to 49 and 5.8% were 50 or older. Among these 4458 HIV‐positive males, 4132 were linked to ART (92.7%).

ITS led to a higher positivity yield (39.9%) in comparison to other community‐based testing (17.7%). In terms of absolute numbers, ITS resulted in a smaller number of men who were newly identified with HIV (n = 2186) in comparison to other testing modalities (n = 2272). Other community testing modalities accounted for 51.0% of HIV‐positive men identified, with ART linkage of 97.3%.

**Figure 4:**
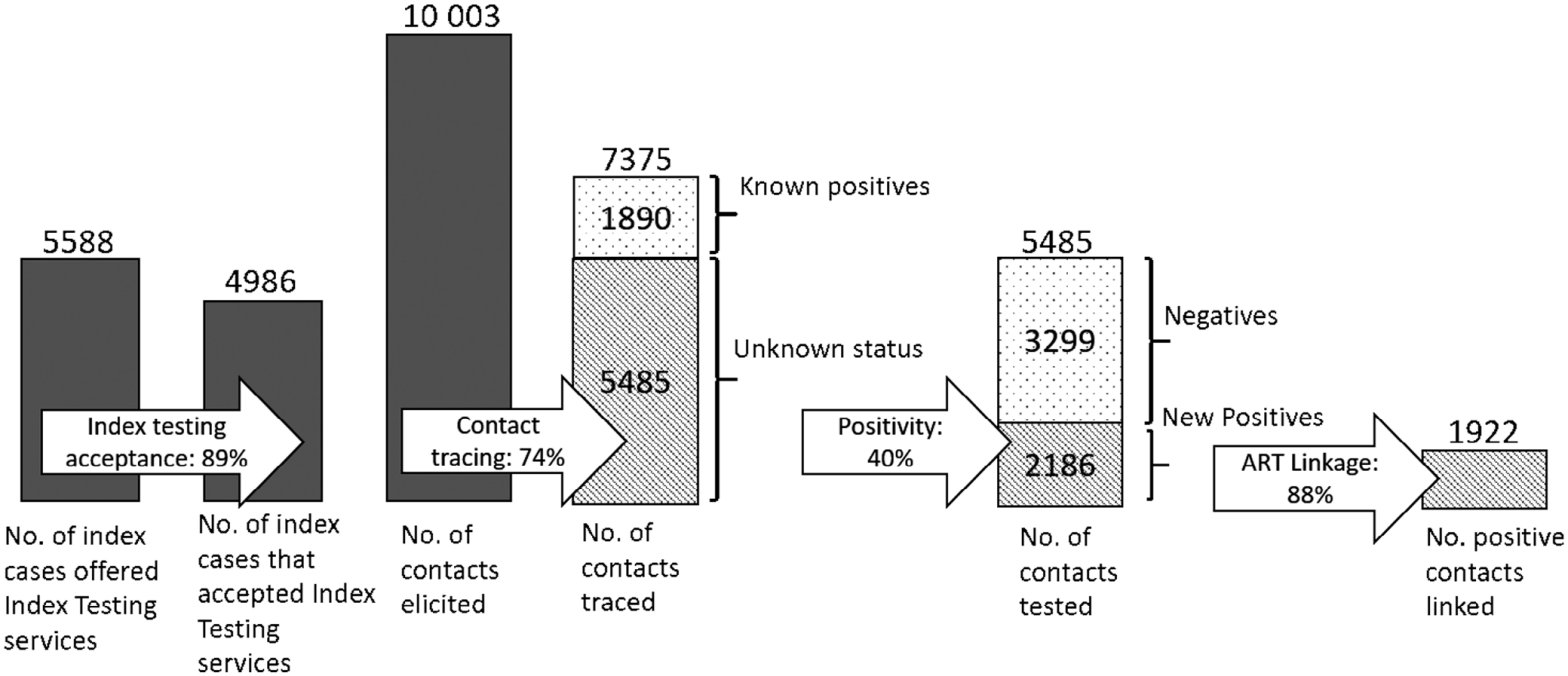
Index cascade for men.

Index testing was offered to 5588 male clients, 89% of whom accepted services. From 10,003 male contacts elicited through index testing, 74% (n = 7375) were traced and all with unknown HIV status (n = 5485) were tested. The positivity yield for men through this modality was 39.9%. Among the 2186 newly identified HIV‐positive clients, 1922 were linked to ART (87.9%) (Figure 4).

## 4 DISCUSSION

We report first year results from CIRKUITS, an innovative community‐based project that effectively engaged men and other key and priority populations with HIV services through a combination of index and targeted community‐based testing coupled with facilitated linkage to HIV prevention and treatment provided by trained CHWs and CLOs. This approach resulted in consistently high numbers of HIV cases identified and high overall testing yield (29%) with robust linkage to ART surpassing 93% among new positives identified. Positivity yield exceeded 20% for both men and women across all districts and age bands, with the exception of 15 to 19‐year‐olds (13%). We found index testing to be a high yield strategy for men, with among the highest positivity yield reported thus far in the literature [20, 21], with a large proportion of cases among young men aged 25 to 39. Targeted community outreach HTS such as mobile and standalone VCT centres were also effective strategies to identify HIV‐positive men in Zambia, resulting in 18% yield, 98% ART linkage and contributing over 50% of all male PLHIV identified, suggesting that a combination of traditional HTS, targeted community and ITS may be needed to reach men. Such high‐yield approaches are critical in light of recent test and treat trials showing that high HIV incidence persisted in SSA communities due to a failure to reach men and key populations with HTS and other HIV services [28, 29, 30].

Despite high overall testing yield across modalities using the CIRKUITS approach, not all community‐based strategies yielded the same results. This observation has been born out elsewhere in the region as detailed in a systematic review examining community and facility‐based HTS in SSA [31]. However, few studies have specifically described results from ITS in SSA, and relatively few reports describe facilitated ART linkage approaches relying on CHWs [31].

We found a high level of acceptance of index testing services. More than 93% of individuals provided information about their sexual partners to enable contact tracing, which we attribute to intensive and continuous mentorship and competency assessment of providers on essential elements of PNS. CHWs were able to elicit nearly two sexual contacts per client. More than three‐quarters of traced contacts were unaware of their HIV status. The ITS modality led to high testing positivity at 45% and ART linkage at 89%, representing a high‐yield entry point into the HIV care cascade.

Other studies from SSA have corroborated findings that index testing is associated with higher HIV positivity yield compared to other testing modalities. Shamu et al. conducted a comparison of index testing to community HTS in South Africa, and found index testing resulted in a positivity yield of 10.3% versus 7.3% [16] for other testing modalities. The ZHCT project in Zimbabwe achieved 32.6% index positivity yield, which was significantly higher than the 4.1% positivity yield observed with facility‐based testing [18].

We found community index testing to be effective at finding HIV‐positive men among elicited sexual contacts. This is consistent with Kenyan data showing that index testing that began with female indexes resulted in significantly higher case‐identification than starting with male indexes [17]. Studies that have focused on HIV‐positive pregnant and breastfeeding women indexes also have reported similarly high positivity yield [32].

In CIRKUITS, we achieved high testing positivity among men primarily by engaging them where they were found, rather than expecting them to come to the facility. Men often perceive health facilities as geared towards women’s and paediatric health, and clinic hours are not favourable to men who often have jobs that prevent them from accessing the clinic [33]. CIRKUITS addressed these barriers by going into the community and providing HIV prevention and testing services in work places, particularly with companies that engage a primarily male workforce, such as construction, security and utility companies. This approach may explain the high absolute number of men who were newly diagnosed with HIV, specifically in workplace and outreach testing settings.

For men, the difference in testing yield was particularly pronounced between community‐based testing (18%) and ITS (40%). Several key elements likely contributed to the higher positivity yield of our index testing approach among men. First, index testing coverage was high as we offered partner elicitation to all clients who tested positive. Second, the elicitation ratio during index testing in the general population was equally high, with a mean of nearly two sexual contacts disclosed per index client. Finally, we maintained high rates of contact tracing, ensuring that nearly all index contacts were followed up and tested.

Interestingly, while index testing resulted in higher yield than other community‐based testing modalities, clients identified via index testing had lower linkage to ART. This result contrasts with other studies which found that linkage was higher among index testing modality versus other approaches [34, 35]. Our finding could be due to facility healthcare workers being present onsite at mobile and community standalone VCT to initiate ART, whereas index contacts in the community were escorted to the facility by CHWs, potentially allowing for greater client attrition before ART start. This finding may also reflect less engagement on the part of the client identified via index testing. Persons reached through workplace testing must still actively present themselves for testing, which may reflect either underlying concerns about their HIV status or their health in general. Clients identified via index testing, however, play a less active role, and thus may be less motivated to engage in ongoing health care. Once a CIRKUITS client was identified as HIV positive, they were accompanied in person to the nearest facility for same‐day ART initiation. If the client declined, they were repeatedly followed up by the CHW until they began treatment. This close follow‐up and longitudinal engagement with a CHW or other peer provider was thought to facilitate effective linkage to care across settings [36].

As we move towards achieving epidemic control, there is ever‐greater need to find, in a targeted and cost‐effective manner, those PLHIV unaware of their status and being left behind by prevailing HTS approaches. High‐yield approaches such as index testing and targeted community‐based testing are strategies to better reach men and other hidden populations; indeed it is why we have highlighted positivity yields as a measure of success. However, to close the gap to the first 95 such modalities must be part of a comprehensive approach to HIV case finding that bridges communities and facilities, and includes the high absolute number of PLHIV found through traditional facility‐based approaches. As noted by De Cock et al., the majority of new PLHIV are still identified via facility‐based approaches, though their positivity yield is often lower. While ITS and targeted community approaches are high yield approaches, they are also costlier and more labour‐intensive, and the absolute numbers of PLHIV found are typically less. Reaching the first 90 will require more testing overall and scale up of *both* facility‐ and community‐based approaches to ensure that all PLHIV are reached and know their status [37].

### 4.1 LIMITATIONS

Our study was limited by the use of routine programme data collected and analysed in aggregate. Therefore, some analyses were not possible, including individual‐level evaluation of testing and clinical outcomes and examination of the association between testing modality and long‐term HIV outcomes, such as retention in care or VL suppression. Aggregate data also limited our ability to examine individual socio‐demographic factors associated with uptake of the HTS modalities studied. We were not able to examine male testing outcomes by CHW gender, which would have been interesting, as prior studies have shown better uptake of HTS by men when offered by male CHWs and HCWs. [38, 39, 40] Due to the aggregate nature of our data, we were unable to perform further analyses to adjust for other potential confounders and clustering by site.

CIRKUITS focused on community‐based HIV testing, and, therefore, we could not compare our results to facility‐based HIV testing. Finally, while CHWs screened for GBV prior to offering ITS, they did not collect data on the number of individuals either declining index testing due to concerns related to GBV, or screening positive for risk of GBV and thus excluded, and as such we cannot make inferences about the effects of GBV on the results observed. Nor were we able to assess negative social outcomes stemming from partner notification, though reassuringly other studies have shown low rates of partner harm following partner notification [41, 42, 43]. Despite these limitations, our evaluation highlights the programmatic relevance of offering ITS and targeted community‐based testing to men. Further research is needed on potential adverse effects of ITS, as well as new strategies to reach men with differentiated HTS modalities.

## 5 CONCLUSIONS

Index testing and targeted community‐based HTS are effective strategies to identify men living with HIV in Zambia. Index testing results in higher yield, but lower linkage and fewer absolute men identified compared to targeted community‐based HTS. In Zambia, there may be up to 400,000 individuals who are currently unaware of their HIV status [4]. Expansion of effective HIV testing modalities such as those we have implemented in CIRKUITS could accelerate the timeline for achieving near‐universal testing coverage and the first 95 of HIV epidemic control.

## COMPETING INTERESTS

The authors declare that they have no conflicts of interest.

## AUTHORS’ CONTRIBUTIONS

All authors contributed to writing, reviewing and editing the manuscript. LM, KS, MM, MH, MCL and CC designed the study; LM, MM, NN, HS, JC, BP, CN, SM, KN, AS, AM, MH and CC designed project concept and implementation; LM, MM, KT, NN, HS, JC, BP, CN, SM, MH and CC contributed to data collection; and KS, NB, MM, KT, MCL, MH and CC contributed to data analysis. All authors have read and approved the final manuscript.
